# Utility of plasma circulating DNA tumor fraction in bone-only metastatic breast cancer: a real-world outcomes study

**DOI:** 10.1007/s10549-025-07740-4

**Published:** 2025-05-30

**Authors:** Gilbert Bader, Julia C. F. Quintanilha, Deloris Veney, Ryon P. Graf, Mia Levy, Lincoln W. Pasquina, Daniel G. Stover

**Affiliations:** 1 The Ohio State University, Columbus, OH USA; 2 Foundation Medicine, Inc., Boston, MA USA

**Keywords:** Circulating tumor DNA, Breast cancer, Bone metastasis, Real-world evidence

## Abstract

**Purpose:**

Bone metastases develop in 50–70% of patients with metastatic breast cancer (MBC), with around one-third having bone as only site of distant disease (bone-only [BO]). Standard imaging is frequently insufficient to track bone metastases. Evidence suggests that circulating tumor DNA (ctDNA) tumor fraction (TF) is prognostic in MBC. We hypothesized that TF would be detectable and prognostic for BO-MBC.

**Methods:**

MBC patients who underwent FoundationOne LiquidCDX comprehensive genomic profiling within 60 days before starting therapy were included. Clinical data was obtained from the nationwide deidentified Flatiron Health/Foundation Medicine Clinico-Genomic Database between 01/2011 and 12/2023.

**Results:**

We identified 778 patients for inclusion: 299 TF < 1% (TF-low), 175 TF 1–10% (TF-intermediate [int]), 304 TF > 10% (TF-high). Of these, 155 had BO-MBC, 622 had non-BO MBC (1 missing metastasis data). Among samples collected prior to first-line therapy (*n* = 256), there was no significant difference in the proportion of patients with detectable ctDNA comparing BO-MBC to non-BO MBC patients (*P* = 1.0). TF was prognostic among patients with BO-MBC: TF-low demonstrated more favorable real-world overall survival (rwOS) relative to TF-int (hazard ratio [HR] 2.19, 95% confidence interval [CI] 1.1–4.35) and TF-high (HR 2.07, 95% CI 1.12–3.82; log-rank *P* = 0.027). Multivariable analyses confirmed the independent and additive association of TF and less favorable rwOS. In multivariable analyses evaluating clinicopathologic factors associated with TF, non-BO metastases were not associated with higher ctDNA TF.

**Conclusion:**

BO-MBC patients are as likely as non-BO-MBC to have detectable ctDNA and TF remains prognostic among BO-MBC patients, with TF < 1% associated with significantly better prognosis.

**Supplementary Information:**

The online version contains supplementary material available at 10.1007/s10549-025-07740-4.

## Introduction

Among the > 168,000 women and men living with metastatic breast cancer (MBC) in the United States (US), bone metastases (mets) are common, developing in 50–70% of MBC patients [1, 2]. Notably, approximately one-third of MBC patients develop bone mets without other (non-bone) mets, which is termed bone-only MBC (BO-MBC) [3, 4]. Bone metastases are associated with significant morbidity, including pain, fractures, and spinal cord compression [5]. There is no consensus regarding optimal response monitoring of BO-MBC [6], and these patients are often excluded from clinical trials because their disease is not measurable in traditional computed tomography (CT) scans. Specifically, the standard approach to assess tumor response in clinical trials uses the Response Evaluation Criteria in Solid Tumors (RECIST) [7]; for bone metastases, only lytic bone metastases with a soft-tissue component > 1 cm are measurable by RECIST. Standard imaging is frequently insufficient to reliably detect and track bone-only progression [3, 4, 6, 8], leaving providers lacking valuable knowledge to inform treatment decisions. Despite the frequency of BO-MBC, few innovative strategies to complement standard imaging have emerged, leaving disease monitoring as a major clinical challenge facing BO-MBC patients and their care teams.

Technological advances have led to the rapid development of minimally invasive cancer liquid biopsy approaches to interrogate the biology of a cancer through blood analysis [9]. Circulating tumor DNA (ctDNA) analysis has emerged with widespread clinical adoption, regulatory approval of multiple multi-gene assays [10, 11], and incorporation into clinical practice guidelines for advanced cancer management [12, 13]. Levels of ctDNA shed by a given cancer can be variable over time and can be influenced by cancer stage and therapy, yet the tumor fraction (TF) of ctDNA has been consistently found to be prognostic independent of clinicopathological factors in breast and other cancers [9, 14–17].

In this study, we address the hypothesis that ctDNA TF could offer a relevant minimally invasive biomarker for BO-MBC and potentially address important clinical gaps. Specifically, this study aimed to (1) evaluate differences in TF distribution between patients with BO-MBC and non-BO MBC; (2) investigate the prognostic value of TF near the time of therapy initiation; and (3) determine baseline lab and clinical factors associated with TF in BO-MBC and non-BO MBC. The primary outcomes were ctDNA detectability prior to first-line metastatic therapy and association of TF with survival among patients with BO-MBC.

## Methods

### Study population

This study included patients with MBC who underwent genomic testing using liquid comprehensive genomic profiling (CGP) assays at Foundation Medicine during routine care. Samples were collected up to 60 days before the start of therapy. For patients with multiple eligible lines of therapy, where serial liquid samples were collected within 60 days of different therapy lines, the earliest line was used. Clinical data were obtained from the nationwide (US-based) deidentified Flatiron Health and Foundation Medicine Clinico-Genomic Database (FH-FMI CGDB) for breast cancer between January 2011 and December 2023. Retrospective deidentified longitudinal clinical data were derived from electronic health records (EHRs), comprising patient-level structured and unstructured data, and curated via technology-enabled abstraction. Clinical data included demographics, clinical and laboratory features, time of therapy exposure, and survival. These were linked to genomic data derived from FMI CGP tests by deidentified, deterministic matching [18]. The data are deidentified and subject to obligations to prevent reidentification and protect patient confidentiality.

### Comprehensive genomic profiling

Genomic alterations were identified via CGP of > 300 cancer-related genes on FMI's next-generation sequencing (NGS) test (FoundationOne®Liquid CDx [F1LCDx]) [19–21]. Assays were performed on patient liquid specimens in a Clinical Laboratory Improvement Amendments (CLIA)-certified, College of American Pathologists (CAP)-accredited laboratory (Foundation Medicine, Inc.). The level of ctDNA shed in the F1LCDx assay for each specimen was quantified by calculating the ctDNA TF. This was done by combining multiple methods: for samples with significant aneuploidy, the purity assessment from a robust copy-number model that accounts for both the observed coverage variation and allele frequencies of genome-wide single nucleotide polymorphism (SNP) allele frequencies was used to determine the ctDNA TF estimate [22, 23]. When significant aneuploidy was not present, the allele frequencies of short variants and rearrangements deemed very likely to be somatic were used to estimate ctDNA TF [24, 25].

### Outcomes

Real-world overall survival (rwOS) was calculated from the start of treatment to death from any cause, and patients with no record of mortality were right-censored at the date of the last clinic visit or structured EHR activity. rwOS risk intervals were left truncated to the date of the CGP report to account for immortal time, as patients cannot enter the database until a CGP report is provided [26, 27]. The mortality information in the FH-FMI CGDB is a composite derived from deidentified patient‐level data within the EHR, the public Social Security Death Index, and a commercial death dataset mining data from obituaries and funeral homes. This mortality information has been externally validated in comparison to the National Death Index [28]. In addition, the FH-FMI CGDB has replicated associations with survival observed in biomarker subgroup analyses of randomized controlled trials [29, 30].

### Data analysis

rwOS was compared between ctDNA TF groups (< 1% vs. 1–10% vs. > 10%) by Cox models and stratified by BO and non-BO (with or without bone) MBC. Multivariable analyses adjusting for baseline laboratory (lab) and clinical factors—including age, Eastern Cooperative Oncology Group (ECOG) score, race/ethnicity, hormone receptor statuses, histology, line of therapy, practice type, stage at diagnosis, adjuvant therapy, menopausal status, number of metastases, albumin, alkaline phosphatase (ALK), serum creatinine, hemoglobin, lactate dehydrogenase (LDH), and neutrophil-to-lymphocyte ratio (NLR)—were performed. Chi-squared and Wilcoxon rank sum tests were used to assess differences between groups of categorical and continuous variables. To evaluate the primary endpoint of differences in TF distribution between patients with BO-MBC and non-BO MBC, based on the available samples size of 777, an alpha level of 0.05 and two-sample proportion test of unequal sample sizes, the power of the analysis is 63%. The Cohen's d effect size is 0.21. That means our sample size had a moderate power (63% at *α* = 0.05) to detect a projected effect size of 0.21 (small effect size)**.** The association between ctDNA TF and baseline lab and clinical features was performed using linear regression, with ctDNA TF log_2_(*x* + 1) transformed to avoid log_2_(0). Missing values in variables with < 20% missingness were imputed from random forest models trained on the observed covariates using the R package *missForest*. Variables with greater degrees of missingness had missing values treated as a separate category. R version 4.3.1 software (R Project for Statistical Computing, RRID:SCR_001905) was used for all statistical analyses.

## Results

**Fig. 1:**
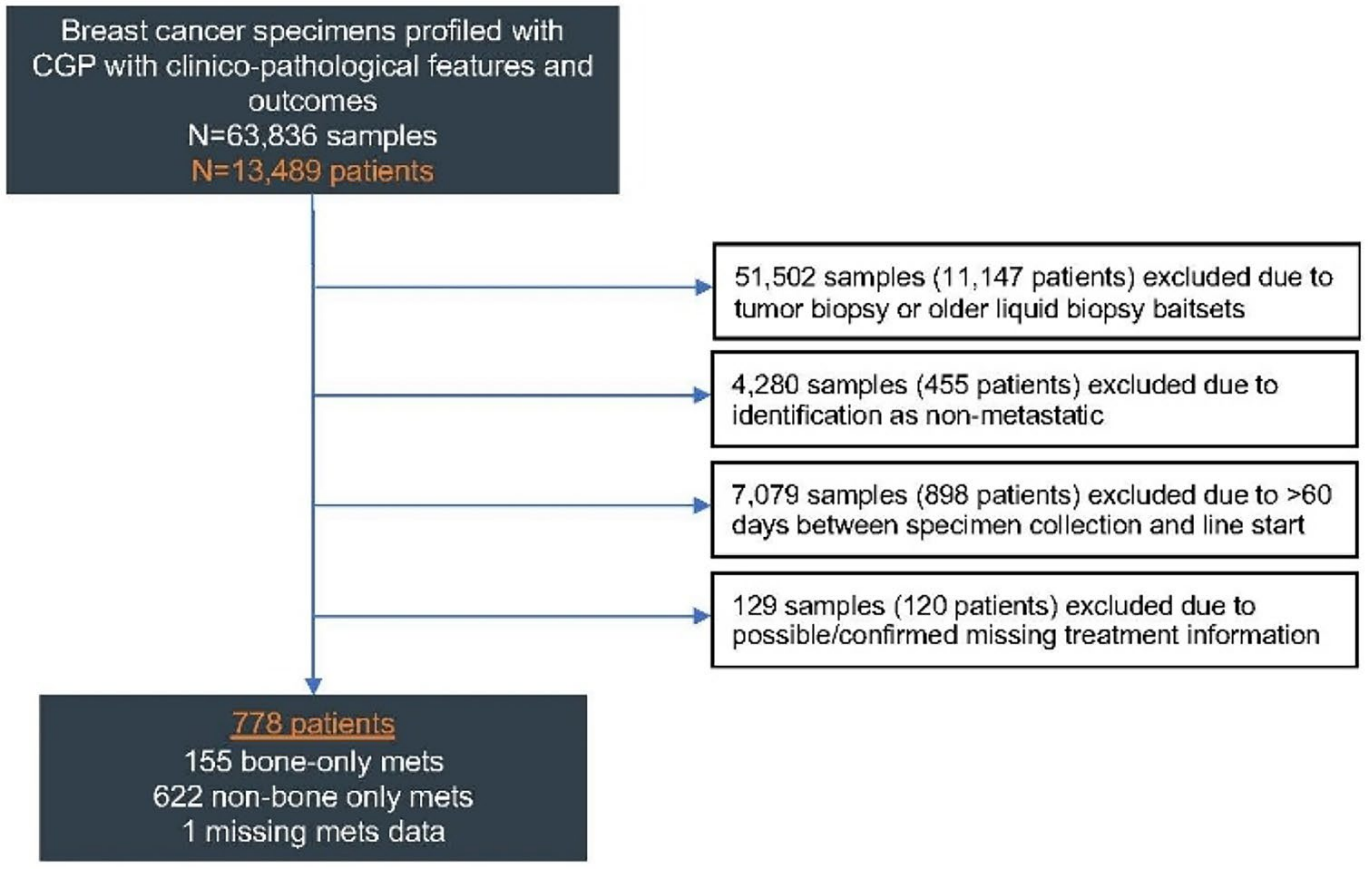
Cohort selection. Samples from patients with a breast cancer diagnosis submitted for CGP at Foundation Medicine between January 2011 and December 2023 were considered for analysis. Samples were excluded if from tumor biopsy or older baitset, identified as non-metastatic, collected > 60 days prior to the start of therapy, or possible/confirmed missing treatment information. *CGP* comprehensive genomic profiling, *LBx* liquid biopsy, *LOT* line of therapy, *TBx* tissue biopsy, *mets* metastasis

A total of 778 patients were included in the study: 155 with BO-MBC, 622 with non-BO MBC, and 1 with missing metastasis site data (Fig. 1).

Of these, 299 had ctDNA TF < 1% (TF-low), 175 had ctDNA 1–10% (TF-intermediate [int]) and 304 had ctDNA TF > 10% (TF-high). The baseline characteristics of MBC patients by ctDNA TF status are described in Table 1. As anticipated, higher ctDNA TF was significantly associated with greater disease burden, defined as 2 + sites of metastases (*P* < 0.001) and estrogen receptor (ER)-negative disease (*P* = 0.007). In addition, higher ctDNA was associated with worse performance status/ECOG score of 2 + (*P* < 0.001), albumin below the lower limit of normal (*P* = 0.047), anemia (*P* = 0.012), elevation in ALK (*P* < 0.001), and higher NLR (*P* = 0.006). Although histology was not available for most patients, there was no significant difference between histology, with invasive ductal carcinoma (IDC) and invasive lobular carcinoma (ILC) having similar proportions of patients with TF-low (36.7% IDC vs. 40.3% ILC, respectively), TF-int (22.9% IDC vs. 23.9% ILC, respectively), and TF-high (40.2% IDC vs. 35.8% ILC, respectively). Supplemental Tables S1 and S2 show baseline characteristics for MBC patients with BO and non-BO metastasis, respectively.

**Table 1:** Baseline characteristics of patients by circulating DNA tumor fraction status

	TF-low (ctDNA TF < 1%) (*n* = 299)	TF-int (ctDNA TF 1–10%) (*n* = 175)	TF-high (ctDNA TF10%+) (*n* = 304)	Total (*N* = 778)	*P* value
Age					0.066
Median (Q1, Q3)	61.0 (53.0, 69.0)	62.0 (53.0, 72.0)	60.0 (51.0, 67.0)	61.0 (52.0, 69.0)	
Gender					0.852
Female	297 (99.3%)	173 (98.9%)	301 (99.0%)	771 (99.1%)	
Male	2 (0.7%)	2 (1.1%)	3 (1.0%)	7 (0.9%)	
ECOG PS					< 0.001
0	128 (50.6%)	60 (40.0%)	94 (34.6%)	282 (41.8%)	
1	109 (43.1%)	65 (43.3%)	123 (45.2%)	297 (44.0%)	
2 +	16 (6.3%)	25 (16.7%)	55 (20.2%)	96 (14.2%)	
Missing	46	25	32	103	
Race/ethnicity					0.738
Hispanic or Latino	13 (4.3%)	7 (4.0%)	17 (5.6%)	37 (4.8%)	
Not Hispanic or Latino/Black or African American	35 (11.7%)	22 (12.6%)	38 (12.5%)	95 (12.2%)	
Not Hispanic or Latino/other or multiple races	21 (7.0%)	18 (10.3%)	18 (5.9%)	57 (7.3%)	
Not Hispanic or Latino/unknown race	10 (3.3%)	4 (2.3%)	10 (3.3%)	24 (3.1%)	
Not Hispanic or Latino/White	166 (55.5%)	98 (56.0%)	158 (52.0%)	422 (54.2%)	
Unknown ethnicity	54 (18.1%)	26 (14.9%)	63 (20.7%)	143 (18.4%)	
ER status					0.007
Negative	48 (16.1%)	39 (22.3%)	81 (26.6%)	168 (21.6%)	
Positive	251 (83.9%)	136 (77.7%)	223 (73.4%)	610 (78.4%)	
PR status					0.343
Negative	129 (43.1%)	79 (45.1%)	149 (49.0%)	357 (45.9%)	
Positive	170 (56.9%)	96 (54.9%)	155 (51.0%)	421 (54.1%)	
HER2 status					0.792
Negative	265 (89.2%)	157 (90.8%)	269 (88.8%)	691 (89.4%)	
Positive	32 (10.8%)	16 (9.2%)	34 (11.2%)	82 (10.6%)	
N-miss	2	2	1	5	
Line of therapy					0.207
1	98 (32.8%)	46 (26.3%)	112 (36.8%)	256 (32.9%)	
2	67 (22.4%)	44 (25.1%)	55 (18.1%)	166 (21.3%)	
3	48 (16.1%)	26 (14.9%)	40 (13.2%)	114 (14.7%)	
4 +	86 (28.8%)	59 (33.7%)	97 (31.9%)	242 (31.1%)	
Practice type					0.368
Academic	45 (15.1%)	29 (16.6%)	33 (10.9%)	107 (13.8%)	
Academic/community	4 (1.3%)	1 (0.6%)	4 (1.3%)	9 (1.2%)	
Community	250 (83.6%)	145 (82.9%)	266 (87.8%)	661 (85.1%)	
Missing	0	0	1	1	
Stage at diagnosis					0.268
Stage I	42 (14.0%)	30 (17.1%)	46 (15.1%)	118 (15.2%)	
Stage II	75 (25.1%)	47 (26.9%)	85 (28.0%)	207 (26.6%)	
Stage III	64 (21.4%)	38 (21.7%)	61 (20.1%)	163 (21.0%)	
Stage IV	82 (27.4%)	46 (26.3%)	95 (31.2%)	223 (28.7%)	
Unknown/not documented	36 (12.0%)	14 (8.0%)	17 (5.6%)	67 (8.6%)	
Adjuvant therapy					0.429
Adjuvant chemo	36 (11.8%)	15 (8.6%)	25 (8.4%)	76 (9.8%)	
Adjuvant ET	89 (29.3%)	55 (31.4%)	82 (27.4%)	226 (29.0%)	
Adjuvant other	12 (3.9%)	3 (1.7%)	8 (2.7%)	23 (3.0%)	
None/unknown	167 (54.9%)	102 (58.3%)	184 (61.5%)	453 (58.2%)	
Histology					0.888
IDC	145 (79.7%)	90 (78.3%)	158 (81.4%)	393 (80.0%)	
ILC	27 (14.8%)	16 (13.9%)	24 (12.4%)	67 (13.6%)	
Other/unknown	10 (5.5%)	9 (7.8%)	12 (6.2%)	31 (6.3%)	
Missing	117	60	110	287	
Menopausal status					0.173
Postmenopausal	96 (49.5%)	62 (53.9%)	88 (48.4%)	246 (50.1%)	
Premenopausal	69 (35.6%)	27 (23.5%)	58 (31.9%)	154 (31.4%)	
Unknown/not documented	29 (14.9%)	26 (22.6%)	36 (19.8%)	91 (18.5%)	
Missing	110	60	117	287	
Number of met sites					< 0.001
1	153 (51.2%)	61 (34.9%)	81 (26.6%)	295 (37.9%)	
2 +	146 (48.8%)	114 (65.1%)	223 (73.4%)	483 (62.1%)	
Metastasis site					0.056
Bone only	72 (24.2%)	33 (18.9%)	50 (16.4%)	155 (19.9%)	
Non-bone only	226 (75.8%)	142 (81.1%)	254 (83.6%)	622 (80.1%)	
Missing	1	0	0	1	
Albumin					0.047
< LLN	28 (10.6%)	24 (15.4%)	52 (17.9%)	104 (14.6%)	
≥ LLN	237 (89.4%)	132 (84.6%)	238 (82.1%)	607 (85.4%)	
Missing	34	19	14	67	
ALK					< 0.001
≤ ULN	233 (87.6%)	122 (77.7%)	150 (51.5%)	505 (70.7%)	
> ULN	33 (12.4%)	35 (22.3%)	141 (48.5%)	209 (29.3%)	
Missing	33	18	13	64	
Serum creatinine					0.603
≤ ULN	222 (86.0%)	127 (82.5%)	238 (85.3%)	587 (84.9%)	
> ULN	36 (14.0%)	27 (17.5%)	41 (14.7%)	104 (15.1%)	
Missing	41	21	25	87	
Hemoglobin					0.012
< LLN	108 (39.9%)	71 (44.4%)	153 (52.2%)	332 (45.9%)	
≥ LLN	163 (60.1%)	89 (55.6%)	140 (47.8%)	392 (54.1%)	
Missing	28	15	11	54	
LDH					0.098
≤ ULN	35 (55.6%)	19 (57.6%)	28 (39.4%)	82 (49.1%)	
> ULN	28 (44.4%)	14 (42.4%)	43 (60.6%)	85 (50.9%)	
Missing	236	142	233	611	
NLR					0.006
≤ 2.5	100 (43.7%)	55 (41.7%)	75 (30.2%)	230 (37.8%)	
> 2.5	129 (56.3%)	77 (58.3%)	173 (69.8%)	379 (62.2%)	
Missing	70	43	56	169	

### ctDNA detectability in BO-MBC

**Fig. 2:**
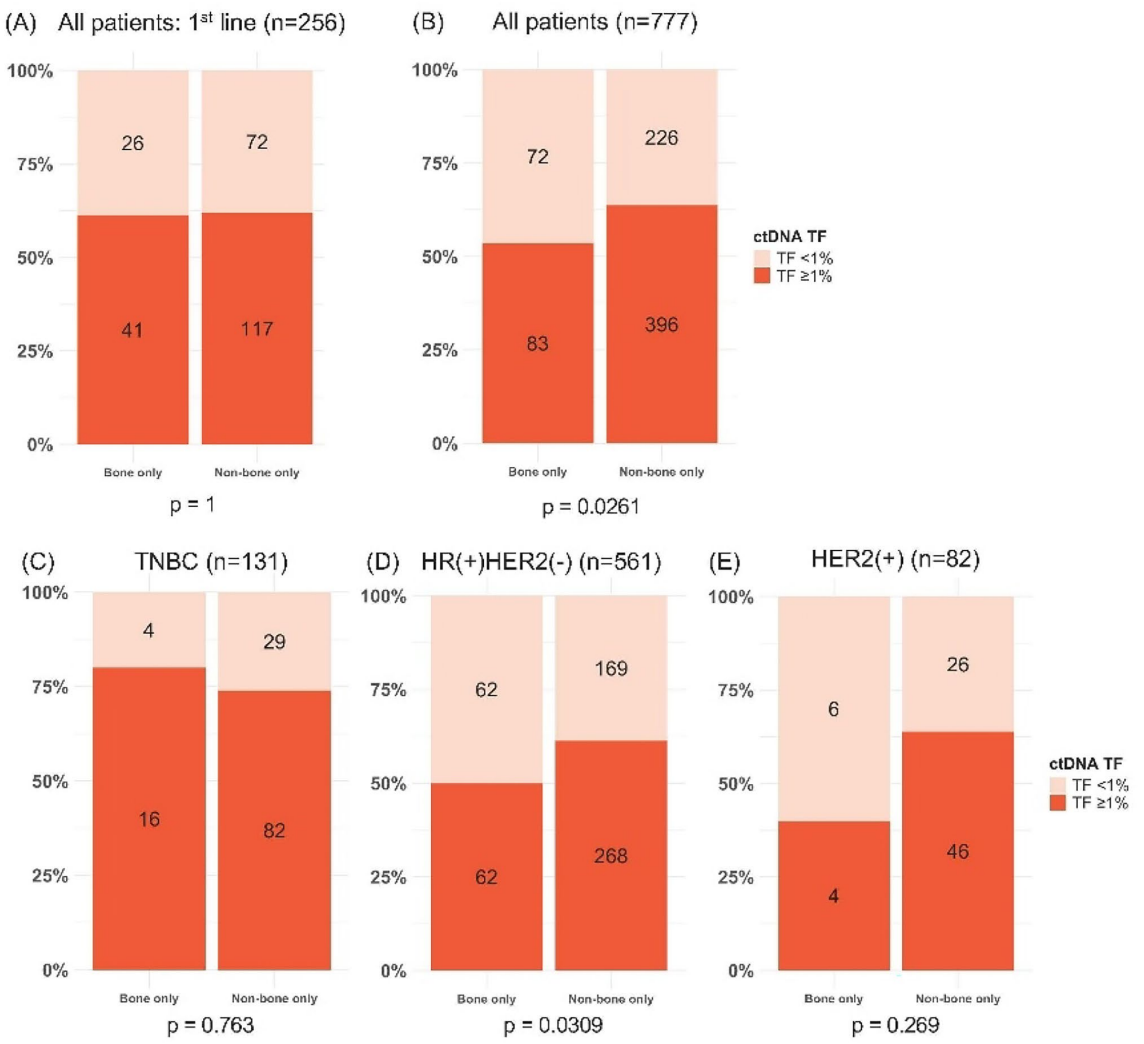
Circulating DNA tumor fraction distribution in patients with bone-only vs. non-bone only metastatic breast cancer (MBC). **A** All patients, restricted to samples collected at first line; **B** all patients; **C** patients with triple-negative breast cancer (TNBC), defined as estrogen and progesterone receptor (hormone receptor)-negative and HER2-negative; **D** patients with hormone receptor-positive/HER2-negative MBC; **E** patients with HER2-positive MBC

While the amount of ctDNA is correlated with metastatic disease burden—in this study and others (reviewed in [31])—we hypothesized that patients with BO-MBC would be as likely as those with non-BO MBC to have detectable ctDNA TF. As our primary endpoint, we evaluated samples collected up to 60 days prior to first-line therapy, a consistent collection timepoint and when ctDNA TF levels may be expected to be highest. We observed that 61.2% of BO-MBC patients and 61.9% of patients with non-BO MBC had ctDNA TF ≥ 1% (*P* = 1.0; Fig. 2A). Evaluating all specimens collected up to 60 days prior to the time of therapy initiation, we observed that 53.5% of MBC patients with BO-MBC and 63.7% of patients with non-BO MBC had ctDNA TF ≥ 1% (*P* = 0.0261, Fig. 2B), suggesting possible modest numerical but statistically significant differences in TF in the context of later lines of therapy. Distribution of ctDNA TF in BO-MBC versus non-BO MBC was similar by MBC receptor subtypes (Fig. 2C–E), with hormone receptor-positive/HER2-negative breast cancer reflecting a slightly higher proportion of samples with TF > 1% in non-BO MBC (50% TF > 1% for BO-MBC vs. 61.3% for non-BO MBC; *P* = 0.03).

**Fig. 3:**
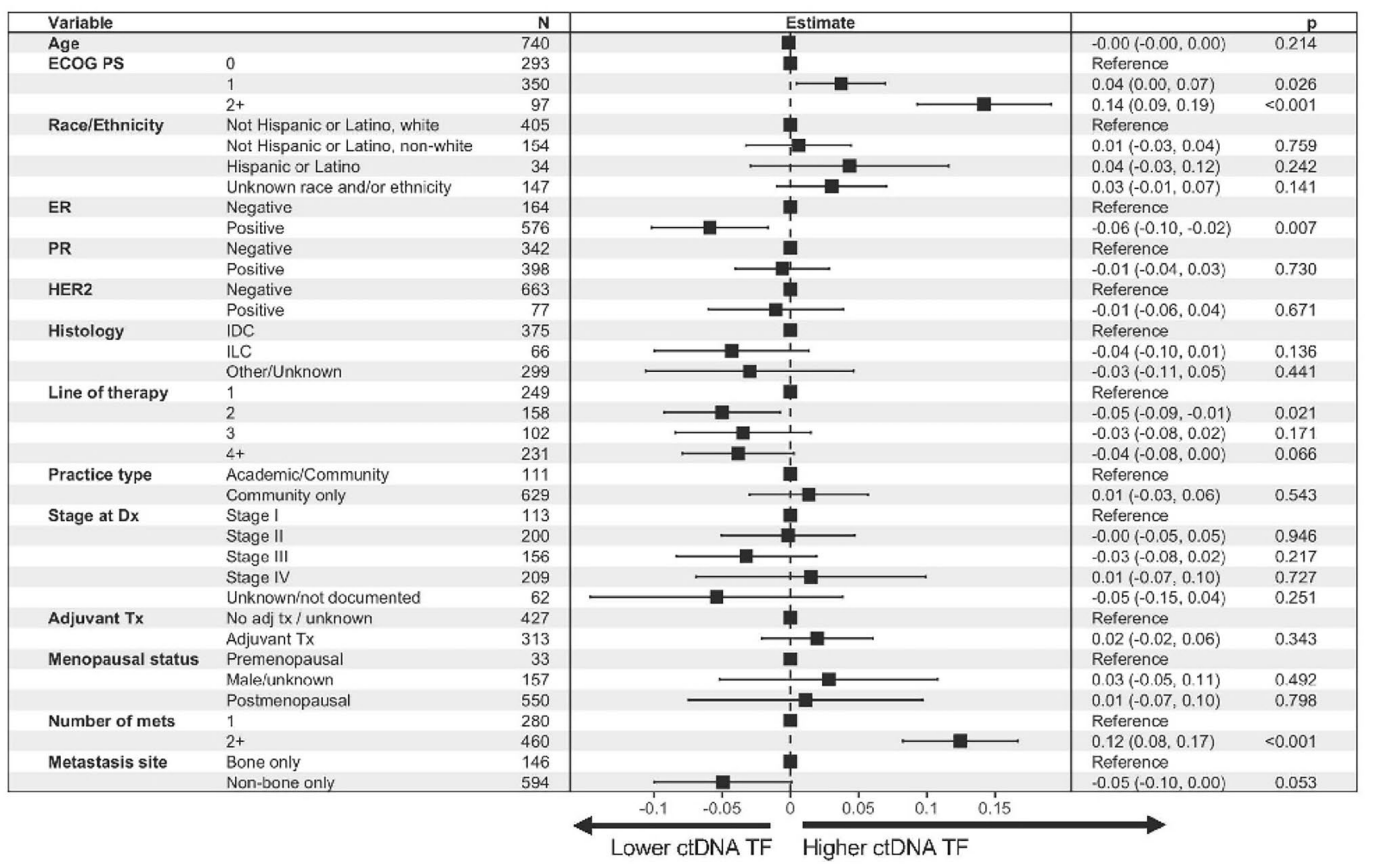
Clinical features associated with circulating DNA tumor fraction. Point estimates and confidence intervals are shown relative to average in the cohort, with estimates to the right of center indicating higher than average TF values and left of center indicating TF values lower than average. Analysis excluded patients with TF status detected but not estimated (*n* = 38). ctDNA TF was log_2_(*x* + 1) transformed to avoid log_2_(0). *ALK* alkaline phosphatase, *ctDNA TF* circulating tumor DNA tumor fraction, *ECOG PS* Eastern Cooperative Oncology Group performance score, *ER* estrogen receptor, *IDC* invasive ductal carcinoma, *ILC* invasive lobular carcinoma, *LLN* lower limit of normal, *LDH* lactate dehydrogenase, *NLR* neutrophil-to-lymphocyte ratio, *PR* progesterone receptor, *ULN* upper limit of normal

As a sensitivity analysis, we also evaluated the distribution of ctDNA TF by 4 groups—ctDNA TF not detectable, TF-low (detectable but < 1%), TF-int, and TF-high—and found no significant difference in any groups (all *P* > 0.1; Supp Fig. S1). In multivariate modeling controlling for clinicopathologic factors, higher ctDNA TF was associated with ECOG performance status ≥ 1 and 2 + sites of metastases. Lower ctDNA TF was associated with ER+ disease (Fig. 3). When adding laboratory values to the model, higher ctDNA TF was associated with elevation of ALK or LDH (Supplemental Fig. S2). Importantly, in both multivariable models, the presence of non-bone metastases was not associated with lower ctDNA TF.

### *Prognostic association of ctDNA TF among patients with BO*-*MBC*

**Fig. 4:**
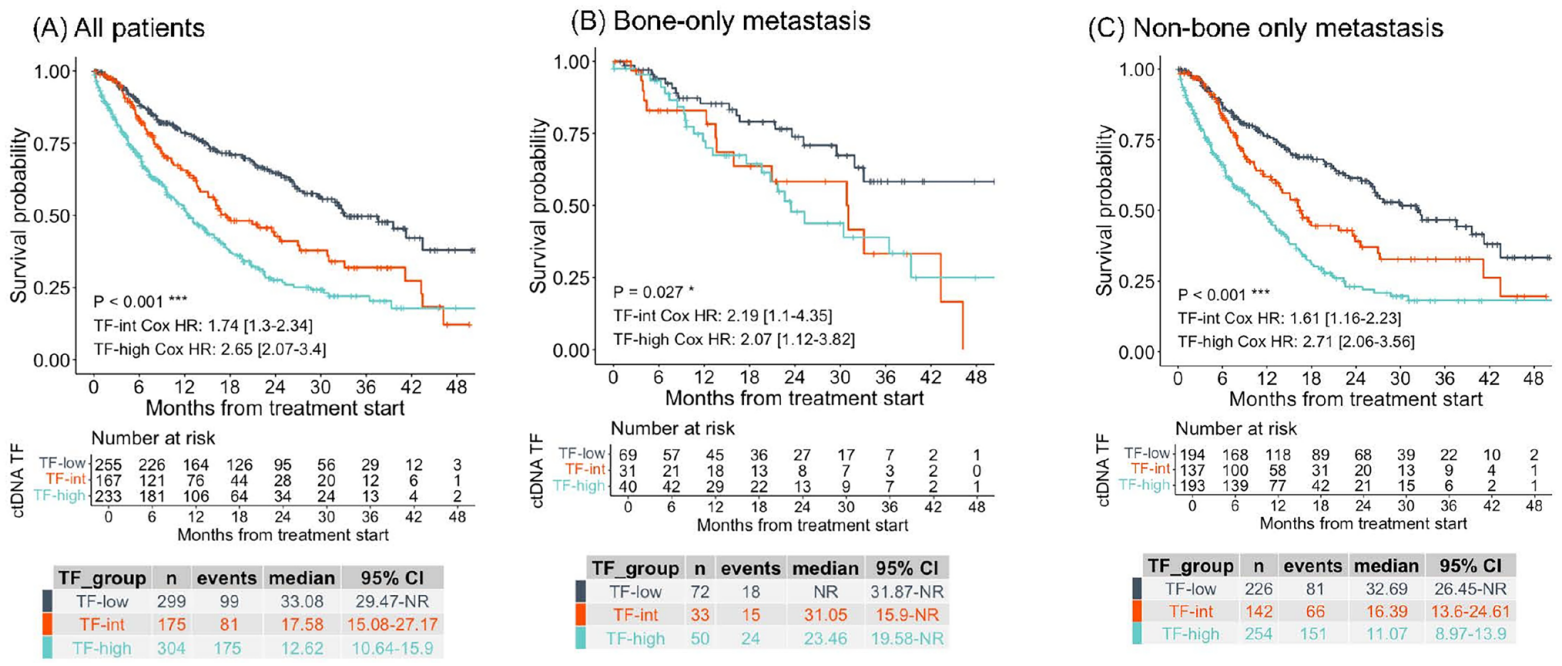
Real-world overall survival for metastatic breast cancer patients by circulating DNA tumor fraction status. **A** All patients; **B** patients with bone-only metastasis; **C** patients with visceral metastasis. *CI* confidence interval, *ctDNA TF* circulating tumor DNA tumor fraction, *HR* hazard ratio, *NR* not reached

ctDNA TF has proven to be prognostic for MBC [9] and among receptor subtypes of MBC such as TNBC [32]. Similarly, in this cohort, compared to patients with ctDNA TF-low (median rwOS 33.08 months), those with ctDNA TF-int had less favorable rwOS (median 17.58 months, hazard ratio [HR] 1.74, 95% confidence interval [CI] 1.3–2.34), as well as those with TF-high (median 12.62 months, HR: 2.65, 95% CI 2.07–3.4) (Fig. 4A). Supplemental Figure S3 shows the results for the same analyses restricted to samples collected prior to the first line of therapy.

To our knowledge, no study has yet evaluated the prognostic association of ctDNA TF in patients with BO-MBC. Among patients with BO-MBC, compared to patients with ctDNA TF-low (median rwOS not reached), those with ctDNA TF-int had less favorable rwOS (median 31.05 months, HR: 2.19, 95% CI 1.1–4.35), as did those with TF-high (median 23.46 months, HR: 2.07, 95% CI 1.12–3.82) (Fig. 4B). Among patients with non-BO MBC, compared to patients with ctDNA TF-low (median rwOS 32.69 months), those with ctDNA TF-high had less favorable rwOS (median 16.39 months, HR: 1.61, 95% CI 1.16–2.23), as did those with TF > 10% (median 11.07 months, HR: 2.17, 95% CI 2.06–3.56) (Fig. 4C).

**Fig. 5:**
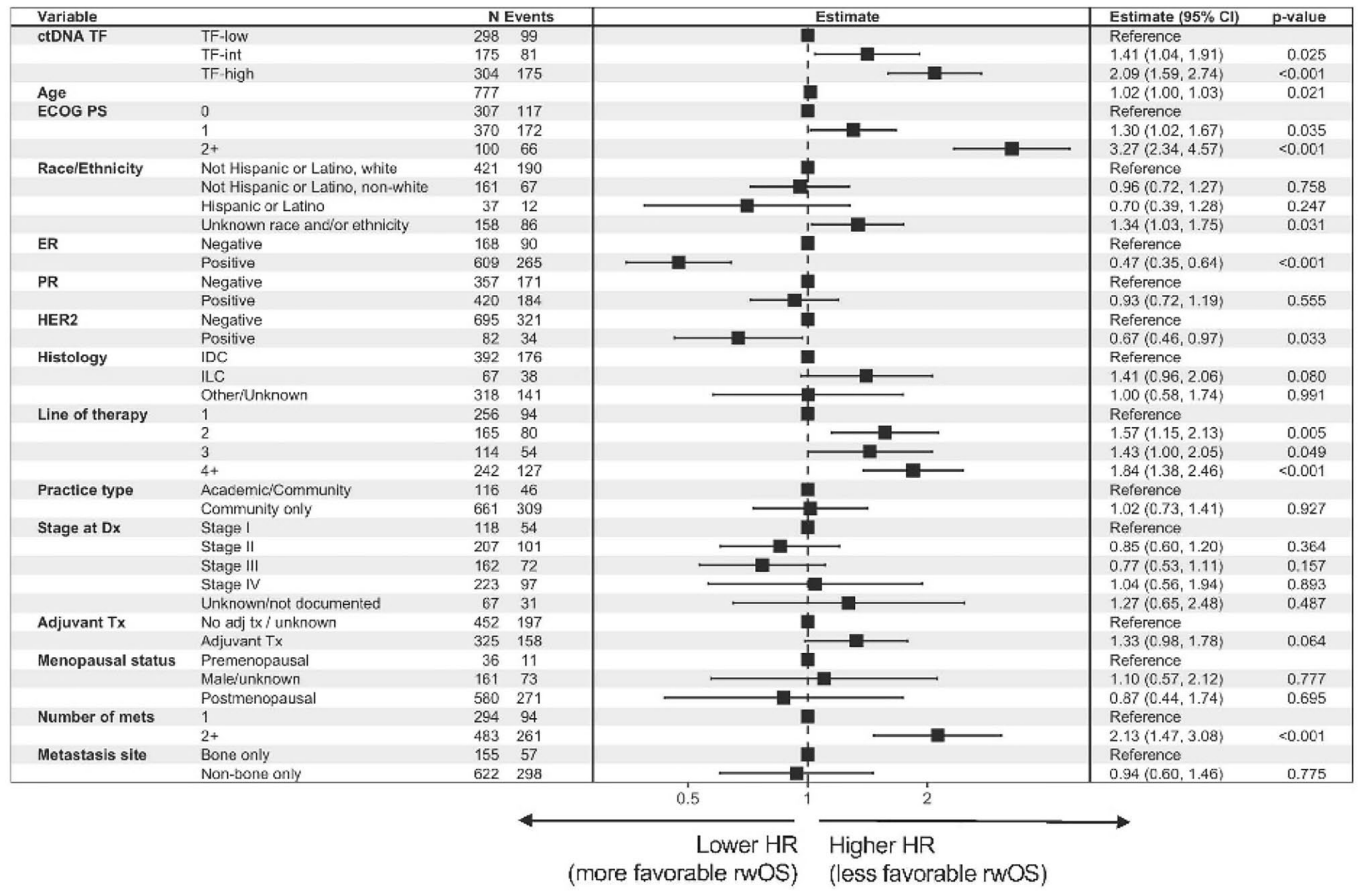
Multivariable model for clinical features associated with real-world overall survival for metastatic breast cancer patients. Point estimates and confidence intervals are shown relative to average in the cohort, with estimates to the right of center indicating hazard ratio greater than one (less favorable rwOS) and left of center indicating hazard ratio less than one (more favorable rwOS). *ALK* alkaline phosphatase, *ctDNA TF* circulating tumor DNA tumor fraction, *ECOG PS* Eastern Cooperative Oncology Group performance score, *ER* estrogen receptor, *IDC* invasive ductal carcinoma, *ILC* invasive lobular carcinoma, *LLN* lower limit of normal, *LDH* lactate dehydrogenase, *NLR* neutrophil-to-lymphocyte ratio, *PR* progesterone receptor, *ULN* upper limit of normal

In multivariate modeling controlling for clinicopathologic factors including BO-MBC versus non-BO MBC, higher ctDNA TF remained independently associated with less favorable rwOS (TF-high, HR: 2.09, 95% CI 1.59–2.74, *P* < 0.001) (Fig. 5). Other factors independently associated with less favorable rwOS include ECOG performance status score 2 + (HR: 3.27, 95% CI 2.34–4.57, *P* < 0.001), 2 + lines of therapy (HR: 1.57, 95% CI 1.15–2.13, *P* = 0.005), and 2 + sites of metastases (HR: 2.13, 95% CI 1.47–3.08, *P* < 0.001). ER-positive (HR: 0.47, 95% CI 0.35–0.64, *P* < 0.001) and HER2-positive disease (HR: 0.67, 65% CI 0.46–0.97, *P* = 0.033) were independently associated with more favorable rwOS (Fig. 5). When adding laboratory values to the model, hypoalbuminemia (HR: 2.16, 95% CI 1.59–2.93, *P* < 0.001), anemia (HR: 1.42, 95% CI 1.13–1.78, *P* = 0.003), and elevation in NLR (HR: 1.44, 95% ci 1.09–1.89, *P* = 0.011) were also independently associated with less favorable rwOS (Supp Figure S4). Among specific mutations, we evaluated *ESR1* mutation status as a common endocrine therapy resistance mechanism with established prognostic significance. we evaluated the distribution of ESR1 mutations in patients with BO vs. non-BO MBC (Supp Figure S5) and, interestingly, do not see a significant difference between bone-only and non-bone only among any group. In addition, we included ESR1 mutation status in the overall multivariable model but did not see a significant additional association of mutation status with survival in the context of other factors (Supp Figure S6).

## Discussion

Metastatic cancer to the bone is common in many cancer types, including breast, prostate, lung, renal cell, and others [33, 34]. Many patients with advanced cancer have bone as the only site of metastasis, including an estimated one-third of patients with MBC [3, 4]. Despite the frequency of BO-MBC as well as the morbidity associated with skeletal events, there remain significant limitations in our ability to reliably detect and track BO progression [3, 4, 6, 8]. Circulating DNA TF is an emerging parameter that has important clinical implications as a prognostic biomarker, and TF dynamics may potentially serve as a predictive biomarker and a marker of treatment response [35]. Although the prevailing thought is that TF is higher with greater metastatic disease burden—particularly non-bone—we found that there was no significant difference in the proportion of patients with BO-MBC versus non-BO MBC starting first-line therapy with a TF ≥ 1%. There were similar numerical proportions of patients among all samples and by receptor subtype. This demonstrates that BO-MBC has detectable ctDNA (based on TF) with comparable frequency to non-BO MBC.

The RECIST criteria remain the standard approach to assess tumor response in clinical trials [7], yet BO-MBC is frequently considered unmeasurable. While many modern breast cancer trials have adapted ways for patients with non-measurable disease to enroll, recently, a RECIST Working Group evaluated ctDNA as a treatment response biomarker in metastatic cancers [36]. Specifically, they noted that RECIST criteria typically require that patients have measurable disease (unlike many BO-MBC patients) and do not provide insight before 6–12 weeks of treatment. As noted in the Working Group report, there are diverse ctDNA assays with varying sensitivity, and TF may have a higher detection threshold than other assay types such as droplet digital polymerase chain reaction (PCR) or personalized assays [36]. More sensitive assays could result in different proportions of ctDNA detectability in BO-MBC versus non-BO MBC.

The reasons why BO-MBC remains challenging to monitor are complex. Innovative imaging offers promise; in particular, nuclear medicine/positron emission tomography (PET)-based imaging is a standard imaging modality for staging cancers that has been investigated as an indicator of therapy response for MBC [37–43]. To detect increasingly subtle changes in tumor response to therapy, PET-based imaging has continued to improve through reduced acquisition time, decreased tracer dose, improved sensitivity, and novel isotopes [44]. Among advanced PET imaging approaches, ^18^F-sodium fluoride PET (Na^18^F-PET) is a high-sensitivity assessment of bone metastases. Na^18^F-PET leverages the natural incorporation of fluoride into bone and offers advantages relative to other methods to assess bone metastases: it can detect both lytic and sclerotic lesions [6, 45] and has greater diagnostic accuracy relative to technetium^99 m^ methylene diphosphonate (^99 m^Tc-MDP) [46–48] or CT [49]. However, Na^18^F-PET has less utility for non-bone disease resulting in limited uptake. There is growing interest in other disease-specific PET tracers including prostate-specific membrane antigen PET (PSMA-PET) and fluoroestradiol PET (FES-PET). Our data raise the question of whether incorporation of ctDNA TF *with* imaging may offer additional utility with standard or PET-based approaches. Importantly, as we consider the incorporation of ctDNA with imaging, the interpretation of changes in ctDNA TF varies—decrease, relative change, “clearance”—and there have been proposed ctDNA-response evaluation criteria in solid tumors [50].

We demonstrated that higher ctDNA TF is associated with worse rwOS among patients with BO-MBC, reinforcing a prognostic association of TF that has been demonstrated in many other contexts. The prognostic association was less strong for BO-MBC relative to non-BO MBC in this study, though BO-MBC had significantly fewer patients (*n* = 140 relative to *n* = 524 for non-BO MBC). When evaluated in multivariable Cox proportional hazard models, the presence of non-BO metastases was not significantly associated with worse rwOS while TF was significantly associated. The interplay between TF and clinicopathologic factors warrants further investigation; for example, laboratory findings including hypoalbuminemia, anemia, and high NLR have been associated with higher TF levels, and each is independently associated with less favorable OS.

As a retrospective real-world study, these analyses have limitations. There is potential for selection bias in the patients for whom F1LCDx was sent—for example, it is possible that patients with a solitary or few bone metastases were less likely to have a sample sent. We also did not compare TF to protein-based tumor markers, although this has been investigated [51]; TF is typically a component of a broader ctDNA-based assay that also reports specific, potentially targetable mutations.

In conclusion, ctDNA TF is detectable in most patients with BO-MBC and at a similar frequency to those with non-BO mets. ctDNA TF appears to be prognostic for BO-MBC. Collectively, this provides reassurance that this emerging biomarker is relevant to BO-MBC patients, a common, yet underserved population. Further studies are needed to investigate its value as a surrogate biomarker, particularly its ability to identify disease progression in concert with standard and emerging imaging modalities.

## Supplementary Information

Below is the link to the electronic supplementary material.

Supplementary file1 (PDF 1210 kb)

## Data Availability

The data that support the findings of this study were originated by and are the property of Flatiron Health, Inc. and Foundation Medicine, Inc., which has restrictions prohibiting the authors from making the dataset publicly available. Requests for data sharing by license or by permission for the specific purpose of replicating results in this manuscript can be submitted to PublicationsDataaccess@flatiron.com and cgdb-fmi@flatiron.com.

## Abbreviations

^99 m^Tc-MDP: Technetium^99 m^ methylene diphosphonate
ALK: Alkaline phosphatase
BO-MBC: Bone-only metastatic breast cancer
CAP: College of American Pathologists
CGP: Comprehensive genomic profiling
CI: Confidence interval
CLIA: Clinical Laboratory Improvement Amendments
CT: Computed tomography
ctDNA: Circulating tumor DNA
ECOG: Eastern Cooperative Oncology Group
EHR: Electronic health record
ER: Estrogen receptor
F1LCDx: FoundationOne Liquid CDx
FES-PET: Fluoroestradiol positron emission tomography
FF-FMI-CGDB: Flatiron Health and Foundation Medicine Clinico-Genomic Database
HR: Hazard ratio
IDC: Invasive ductal carcinoma
ILC: Invasive lobular carcinoma
LDH: Lactate dehydrogenase
MBC: Metastatic breast cancer
mets: Metastases
Na^18^F-PET: ^18^F-sodium fluoride positron emission tomography
NGS: Next-generation sequencing
NLR: Neutrophil-to-lymphocyte ratio
PCR: Polymerase chain reaction
PET: Positron emission tomography
PSMA-PET: Prostate-specific membrane antigen positron emission tomography
RECIST: Response evaluation criteria in solid tumors
rwOS: Real-world overall survival
SNP: Single nucleotide polymorphism
TF: Tumor fraction
US: United States
