# Supplementary material for: Utility of plasma circulating DNA tumor fraction in bone-only metastatic breast cancer: a real-world outcomes study
Source: Breast Cancer Res Treat. 2025 May 30;212(3):475–86. doi: 10.1007/s10549-025-07740-4 (PMC12209397; doi:10.1007/s10549-025-07740-4)
Supplement: Supplementary file 1 — Supplementary file1 (PDF 1210 kb) [file 10549_2025_7740_MOESM1_ESM.pdf]

Supp Table S1. Baseline characteristics of patients with bone-only metastasis by ctDNA TF status.

	TF-low (ctDNA TF <1%) (N=72)	TF-int (ctDNA TF 1-10%) (N=33)	TF-high (ctDNA TF 10%+) (N=50)	Total (N=155)	p value
**Age**					0.651
Median (Q1, Q3)	61.0 (50.8, 71.2)	65.0 (54.0, 73.0)	60.0 (54.2, 68.5)	61.0 (53.0, 71.0)	
**Gender**					-
Female	72 (100.0%)	33 (100.0%)	50 (100.0%)	155 (100.0%)	
**ECOG**					0.017
0	36 (60.0%)	16 (66.7%)	14 (31.8%)	66 (51.6%)	
1	20 (33.3%)	5 (20.8%)	22 (50.0%)	47 (36.7%)	
2+	4 (6.7%)	3 (12.5%)	8 (18.2%)	15 (11.7%)	
Missing	12	9	6	27	
**Race/ethnicity**					0.991
Not Hispanic or Latino / Hispanic or Latino	3 (4.2%)	2 (6.1%)	3 (6.0%)	8 (5.2%)	
Not Hispanic or Latino / Black or African American	11 (15.3%)	3 (9.1%)	6 (12.0%)	20 (12.9%)	
Not Hispanic or Latino / Other or multiple races	6 (8.3%)	2 (6.1%)	3 (6.0%)	11 (7.1%)	
Not Hispanic or Latino / White	40 (55.6%)	19 (57.6%)	28 (56.0%)	87 (56.1%)	
Unknown	12 (16.7%)	7 (21.2%)	8 (16.0%)	10 (20.0%)	
**ER status**					0.138
Negative	6 (8.3%)	7 (21.2%)	9 (18.0%)	22 (14.2%)	
Positive	66 (91.7%)	26 (78.8%)	41 (82.0%)	133 (85.8%)	
**PR status**					0.774
Negative	29 (40.3%)	11 (33.3%)	20 (40.0%)	60 (38.7%)	
Positive	43 (59.7%)	22 (66.7%)	30 (60.0%)	95 (61.3%)	
**HER2 status**					0.601
Negative	66 (91.7%)	31 (96.9%)	47 (94.0%)	144 (93.5%)	
Positive	6 (8.3%)	1 (3.1%)	3 (6.0%)	10 (6.5%)	
Missing	0	1	0	1	
**Line of Therapy**					0.159
1	26 (36.1%)	12 (36.4%)	29 (58.0%)	67 (43.2%)	
2	17 (23.6%)	7 (21.2%)	9 (18.0%)	33 (21.3%)	
3	17 (23.6%)	5 (15.2%)	6 (12.0%)	28 (18.1%)	
4+	12 (16.7%)	9 (27.3%)	6 (12.0%)	27 (17.4%)	
**Practice type**					0.077
Academic	14 (19.4%)	7 (21.2%)	3 (6.0%)	24 (15.5%)	
Community	58 (80.6%)	26 (78.8%)	47 (94.0%)	131 (84.5%)	
**Stage at diagnosis**					0.861
Stage I	14 (19.4%)	5 (15.2%)	6 (12.0%)	25 (16.1%)	
Stage II	14 (19.4%)	9 (27.3%)	10 (20.0%)	33 (21.3%)	
Stage III	15 (20.8%)	6 (18.2%)	13 (26.0%)	34 (21.9%)	
Stage IV	18 (25.0%)	10 (30.3%)	16 (32.0%)	44 (28.4%)	
Unknown/not documented	11 (15.3%)	3 (9.1%)	5 (10.0%)	19 (12.3%)	
**Adjuvant therapy**					0.799
Adjuvant chemo	4 (5.6%)	3 (9.1%)	3 (6.0%)	10 (6.5%)	
Adjuvant ET	22 (30.6%)	13 (39.4%)	18 (36.0%)	53 (34.2%)	
Adjuvant other / None / unknown	46 (63.9%)	17 (51.5%)	29 (58.0%)	92 (59.4%)	
**Histology**					0.786
IDC	26 (59.1%)	14 (70.0%)	18 (62.1%)	58 (62.4%)	
ILC	15 (34.1%)	6 (30.0%)	9 (31.0%)	30 (32.3%)	
Other/unknown	3 (6.8%)	0 (0.0%)	2 (6.9%)	5 (5.4%)	
Missing	28	13	21	62	
**Menopausal status**					0.800
Postmenopausal	24 (54.5%)	12 (60.0%)	15 (51.7%)	51 (54.8%)	
Premenopausal	11 (25.0%)	4 (20.0%)	10 (34.5%)	25 (26.9%)	
Unknown/not documented	9 (20.5%)	4 (20.0%)	4 (13.8%)	17 (18.3%)	
Missing	28	13	21	62	
**Number of met sites**					-
1	72 (100.0%)	33 (100.0%)	50 (100.0%)	155 (100.0%)	
**Metastasis site**					-
Bone only	72 (100.0%)	33 (100.0%)	50 (100.0%)	155 (100.0%)	
**Albumin**					0.204
<LLN	1 (1.7%)	3 (10.3%)	3 (6.5%)	7 (5.2%)	
>=LLN	58 (98.3%)	26 (89.7%)	43 (93.5%)	127 (94.8%)	
Missing	13	4	4	21	
**ALK**					0.001
=<ULN	53 (89.8%)	20 (71.4%)	27 (58.7%)	100 (75.2%)	
>ULN	6 (10.2%)	8 (28.6%)	19 (41.3%)	33 (24.8%)	
Missing	13	5	4	22	
**Serum creatinine**					0.635
=<ULN	48 (84.2%)	22 (75.9%)	37 (82.2%)	107 (81.7%)	
>ULN	9 (15.8%)	7 (24.1%)	8 (17.8%)	24 (18.3%)	
Missing	15	4	5	24	
**Hemoglobin**					0.051
<LLN	17 (27.4%)	13 (41.9%)	23 (50.0%)	53 (38.1%)	
>=LLN	45 (72.6%)	18 (58.1%)	23 (50.0%)	86 (61.9%)	
Missing	10	2	4	16	
**LDH**					0.523
=<ULN	11 (68.8%)	4 (80.0%)	7 (53.8%)	22 (64.7%)	
>ULN	5 (31.2%)	1 (20.0%)	6 (46.2%)	12 (35.3%)	
Missing	56	28	37	121	
**NLR**					0.19
=<2.5	29 (55.8%)	8 (33.3%)	19 (50.0%)	56 (49.1%)	
>2.5	23 (44.2%)	16 (66.7%)	19 (50.0%)	58 (50.9%)	
Missing	20	9	12	41	

ALK: alkaline phosphatase; ctDNA TF: circular tumor DNA Tumor Fraction; ECOG PS: Eastern Cooperative Oncology Group performance score; ER: estrogen receptor; IDC: invasive ductal carcinoma; ILC: invasive lobular carcinoma; LLN = lower limit of normal; LDH: NLR: neutrophil-to-lymphocyte ratio; lactate dehydrogenase; PR: progesterone receptor ULN = upper limits of normal.

Supp Table S2. Baseline characteristics of patients with non-bone only metastasis by ctDNA TF status.

	ctDNA TF<1% (N=226)	ctDNA TF 1-10% (N=142)	ctDNA TF10%+ (N=254)	Total (N=622)	p value
**Age**					0.105
Median (Q1, Q3)	61.0 (53.0, 68.0)	62.0 (53.0, 71.8)	60.0 (51.0, 67.0)	61.0 (52.0, 68.0)	
**Gender**					0.893
Female	224 (99.1%)	140 (98.6%)	251 (98.8%)	615 (98.9%)	
Male	2 (0.9%)	2 (1.4%)	3 (1.2%)	7 (1.1%)	
**ECOG**					< 0.001
0	92 (47.9%)	44 (34.9%)	80 (35.1%)	216 (39.6%)	
1	88 (45.8%)	60 (47.6%)	101 (44.3%)	249 (45.6%)	
2+	12 (6.2%)	22 (17.5%)	47 (20.6%)	81 (14.8%)	
Missing	34	16	26	76	
**Race/ethnicity**					0.347
Hispanic or Latino	10 (4.4%)	5 (3.5%)	14 (5.5%)	29 (4.7%)	
Not Hispanic or Latino / Black or African American	24 (10.6%)	19 (13.4%)	32 (12.6%)	75 (12.1%)	
Not Hispanic or Latino / Other or multiple races	15 (6.6%)	16 (11.3%)	15 (5.9%)	46 (7.4%)	
Not Hispanic or Latino / White	125 (55.3%)	79 (55.6%)	130 (51.2%)	334 (53.7%)	
Unknown	52 (23.0%)	23 (16.2%)	63 (24.8%)	138 (22.2%)	
**ER status**					0.04
Negative	42 (18.6%)	32 (22.5%)	72 (28.3%)	146 (23.5%)	
Positive	184 (81.4%)	110 (77.5%)	182 (71.7%)	476 (76.5%)	
**PR status**					0.359
Negative	100 (44.2%)	68 (47.9%)	129 (50.8%)	297 (47.7%)	
Positive	126 (55.8%)	74 (52.1%)	125 (49.2%)	325 (52.3%)	
**HER2 status**					0.891
Negative	198 (88.4%)	126 (89.4%)	222 (87.7%)	546 (88.3%)	
Positive	26 (11.6%)	15 (10.6%)	31 (12.3%)	72 (11.7%)	
Missing	2	1	1	4	
**Line of Therapy**					0.45
1	72 (31.9%)	34 (23.9%)	83 (32.7%)	189 (30.4%)	
2	49 (21.7%)	37 (26.1%)	46 (18.1%)	132 (21.2%)	
3	31 (13.7%)	21 (14.8%)	34 (13.4%)	86 (13.8%)	
4+	74 (32.7%)	50 (35.2%)	91 (35.8%)	215 (34.6%)	
**Practice type**					0.779
Academic	31 (13.7%)	22 (15.5%)	30 (11.9%)	83 (13.4%)	
Academic/Community	4 (1.8%)	1 (0.7%)	4 (1.6%)	9 (1.4%)	
Community	191 (84.5%)	119 (83.8%)	219 (86.6%)	529 (85.2%)	
Missing	0	0	1	1	
**Stage at diagnosis**					0.258
Stage I	28 (12.4%)	25 (17.6%)	40 (15.7%)	93 (15.0%)	
Stage II	61 (27.0%)	38 (26.8%)	75 (29.5%)	174 (28.0%)	
Stage III	48 (21.2%)	32 (22.5%)	48 (18.9%)	128 (20.6%)	
Stage IV	64 (28.3%)	36 (25.4%)	79 (31.1%)	179 (28.8%)	
Unknown/not documented	25 (11.1%)	11 (7.7%)	12 (4.7%)	48 (7.7%)	
**Adjuvant therapy**					0.532
Adjuvant chemo	21 (9.3%)	12 (8.5%)	33 (13.0%)	66 (10.6%)	
Adjuvant ET	60 (26.5%)	42 (29.6%)	71 (28.0%)	173 (27.8%)	
Adjuvant other / None / unknown	145 (64.2%)	88 (62.0%)	150 (59.1%)	383 (61.6%)	
**Histology**					0.698
IDC	118 (86.1%)	76 (80.0%)	140 (84.8%)	334 (84.1%)	
ILC	12 (8.8%)	10 (10.5%)	15 (9.1%)	37 (9.3%)	
Other/unknown	7 (5.1%)	9 (9.5%)	10 (6.1%)	26 (6.5%)	
Missing	89	47	89	225	
**Menopausal status**					0.404
Perimenopausal	3 (2.2%)	3 (3.2%)	5 (3.0%)	11 (2.8%)	
Postmenopausal	63 (46.0%)	50 (52.6%)	81 (49.1%)	194 (48.9%)	
Premenopausal	47 (34.3%)	23 (24.2%)	59 (35.8%)	129 (32.5%)	
Unknown/not documented	23 (16.8%)	19 (20.0%)	24 (17.5%)	60 (15.1%)	
Missing	89	47	89	225	
**Number of met sites**					< 0.001
1	80 (35.4%)	28 (19.7%)	31 (12.2%)	139 (22.3%)	
2+	146 (64.6%)	114 (80.3%)	223 (87.8%)	483 (77.7%)	
**Metastasis site**					-
Non-bone only	226 (100.0%)	142 (100.0%)	254 (100.0%)	622 (100.0%)	
**Albumin**					0.149
<LLN	27 (13.2%)	21 (16.5%)	49 (20.1%)	97 (16.8%)	
>=LLN	178 (86.8%)	106 (83.5%)	195 (79.9%)	479 (83.2%)	
Missing	21	15	10	46	
**ALK**					< 0.001
=<ULN	179 (86.9%)	102 (79.1%)	123 (50.2%)	404 (69.7%)	
>ULN	27 (13.1%)	27 (20.9%)	122 (49.8%)	176 (30.3%)	
Missing	20	13	9	42	
**Serum creatinine**					0.816
=<ULN	173 (86.5%)	105 (84.0%)	201 (85.9%)	479 (85.7%)	
>ULN	27 (13.5%)	20 (16.0%)	33 (14.1%)	80 (14.3%)	
Missing	26	17	20	63	
**Hemoglobin**					0.109
<LLN	90 (43.3%)	58 (45.0%)	130 (52.6%)	278 (47.6%)	
>=LLN	118 (56.7%)	71 (55.0%)	117 (47.4%)	306 (52.4%)	
Missing	18	13	7	38	
**LDH**					0.188
=<ULN	24 (51.1%)	15 (53.6%)	21 (36.2%)	60 (45.1%)	
>ULN	23 (48.9%)	13 (46.4%)	37 (63.8%)	73 (54.9%)	
Missing	179	114	196	489	
**NLR**					0.002
=<2.5	71 (40.3%)	47 (43.5%)	56 (26.7%)	174 (35.2%)	
>2.5	105 (59.7%)	61 (56.5%)	154 (73.3%)	320 (64.8%)	
Missing	50	34	44	128	

ALK: alkaline phosphatase; ctDNA TF: circular tumor DNA Tumor Fraction; ECOG PS: Eastern Cooperative Oncology Group performance score; ER: estrogen receptor; IDC: invasive ductal carcinoma; ILC: invasive lobular carcinoma; LLN = lower limit of normal; LDH: NLR: neutrophil-to-lymphocyte ratio; lactate dehydrogenase; PR: progesterone receptor ULN = upper limits of normal.

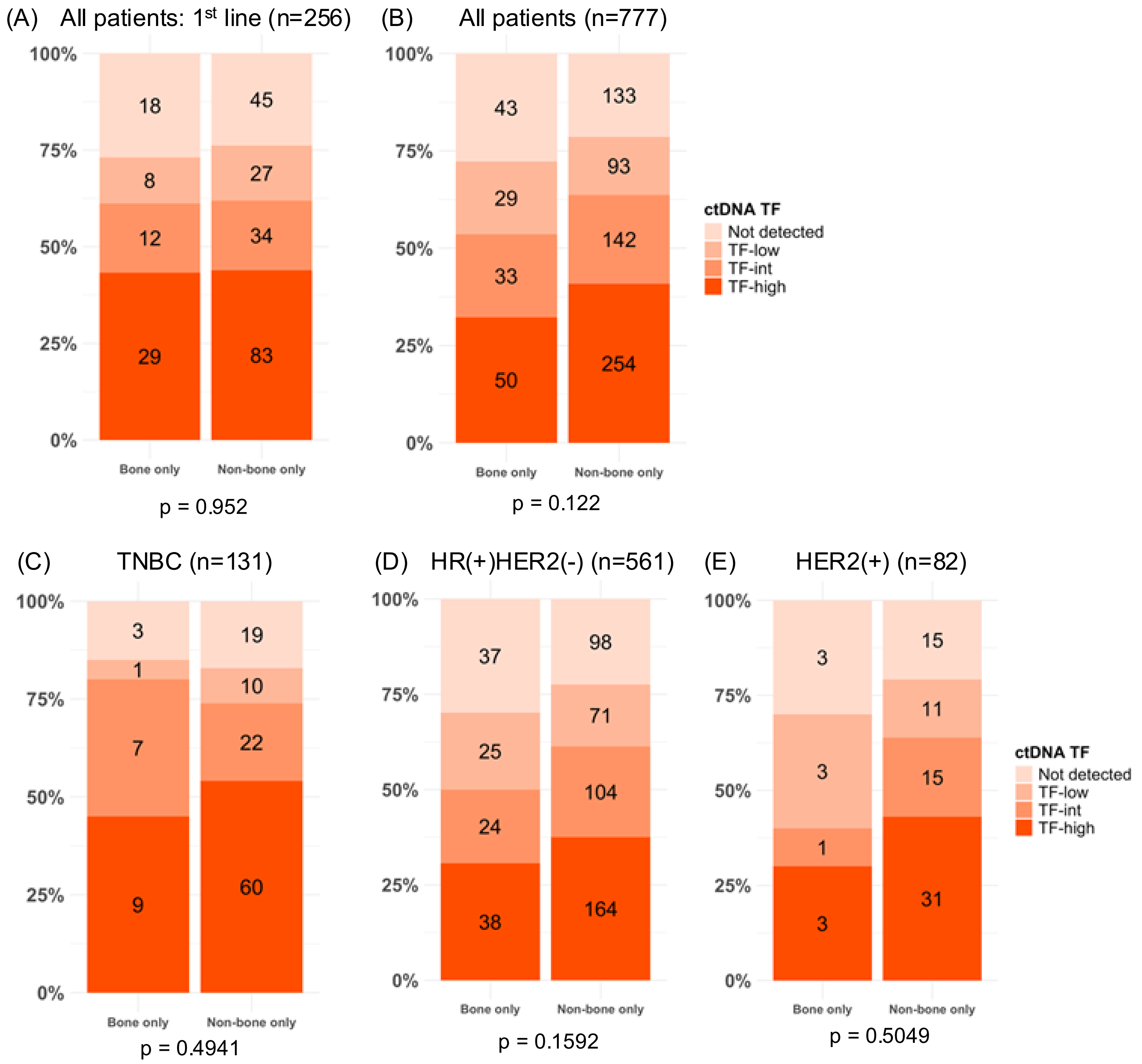

Supp Figure S1. ctDNA TF distribution in patients with bone-only vs. non-bone only metastasis (A) All patients, restricted to samples collected at 1st line; (B) All patients; (C) TNBC patients; (D) HR(+)HER2(-) patients; (E) HER2(+) patients.

Variable		N	Estimate	p
**Age**		740	-0.00 (-0.00, 0.00)	0.579
**ECOG PS**	0	293	Reference	
	1	350	0.04 (0.00, 0.07)	0.025
	2+	97	0.11 (0.06, 0.16)	<0.001
**Race/Ethnicity**	Not Hispanic or Latino, white	405	Reference	
	Not Hispanic or Latino, non-white	154	0.01 (-0.03, 0.04)	0.673
	Hispanic or Latino	34	0.07 (-0.00, 0.13)	0.063
	Unknown race and/or ethnicity	147	0.02 (-0.02, 0.05)	0.407
**ER**	Negative	164	Reference	
	Positive	576	-0.05 (-0.09, -0.01)	0.013
**PR**	Negative	342	Reference	
	Positive	398	0.01 (-0.03, 0.04)	0.757
**HER2**	Negative	663	Reference	
	Positive	77	-0.00 (-0.05, 0.05)	0.995
**Histology**	IDC	375	Reference	
	ILC	66	-0.05 (-0.10, 0.01)	0.100
	Other/Unknown	299	-0.03 (-0.10, 0.04)	0.387
**Line of therapy**	1	249	Reference	
	2	158	-0.03 (-0.08, 0.01)	0.099
	3	102	-0.02 (-0.07, 0.02)	0.347
	4+	231	-0.04 (-0.08, 0.00)	0.056
**Practice type**	Academic/Community	111	Reference	
	Community only	629	0.01 (-0.03, 0.06)	0.536
**Stage at Dx**	Stage I	113	Reference	
	Stage II	200	-0.01 (-0.05, 0.04)	0.732
	Stage III	156	-0.03 (-0.08, 0.02)	0.214
	Stage IV	209	0.02 (-0.06, 0.10)	0.592
	Unknown/not documented	62	-0.03 (-0.12, 0.05)	0.458
**Adjuvant Tx**	No adj tx / unknown	427	Reference	
	Adjuvant Tx	313	0.02 (-0.02, 0.06)	0.304
**Menopausal status**	Premenopausal	33	Reference	
	Male/unknown	157	0.00 (-0.07, 0.08)	0.915
	Postmenopausal	550	-0.01 (-0.10, 0.07)	0.734
**Number of mets**	1	280	Reference	
	2+	460	0.09 (0.05, 0.13)	<0.001
**Metastasis site**	Bone only	146	Reference	
	Non-bone only	594	-0.04 (-0.09, 0.01)	0.114
**Albumin**	>=LLN	638	Reference	
	<LLN	102	0.04 (-0.00, 0.09)	0.056
**ALK**	=<ULN	521	Reference	
	>ULN	219	0.13 (0.10, 0.16)	<0.001
**Serum creatinine**	=<ULN	638	Reference	
	>ULN	102	-0.01 (-0.06, 0.03)	0.523
**Hemoglobin**	>=LLN	416	Reference	
	<LLN	324	0.02 (-0.01, 0.05)	0.230
**LDH**	=<ULN	77	Reference	
	>ULN	81	0.10 (0.04, 0.16)	0.002
	Unknown	582	0.03 (-0.01, 0.08)	0.164
**NLR**	=<2.5	214	Reference	
	>2.5	363	0.02 (-0.02, 0.05)	0.285
	Unknown	163	0.01 (-0.03, 0.06)	0.603

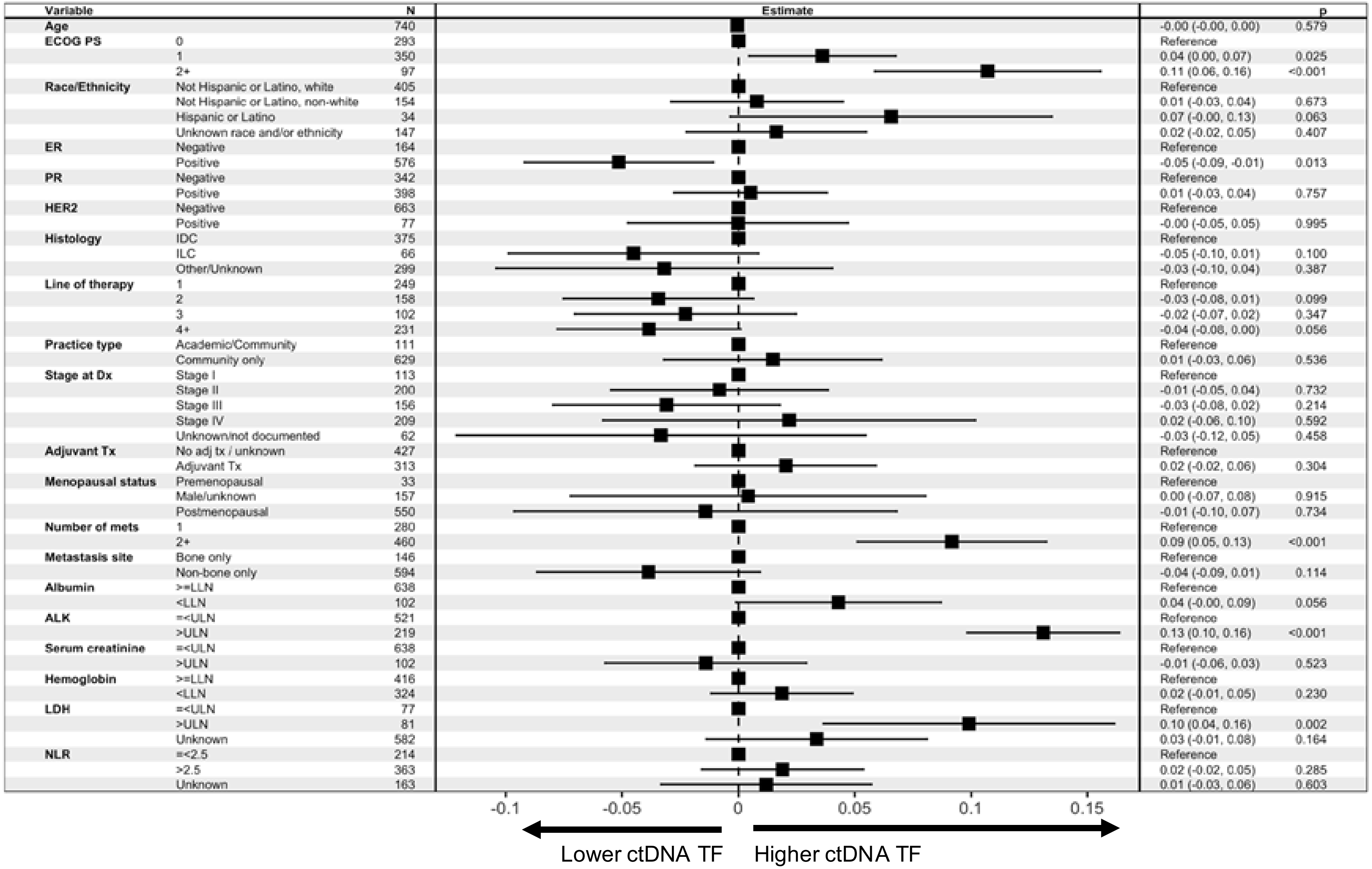

Supp Figure S2. Clinical and laboratory features associated with ctDNA TF.

Point estimates and confidence intervals are shown relative to average in the cohort, with estimates to the right of center indicating higher than average TF values, left of center indicating TF values lower than average. Analysis excluded patients with TF status detected but not estimated value (n=38). ctDNA TF was $\log_2(x + 1)$ transformed to avoid $\log_2(0)$. Variables with less than 20% missingness were imputed, while variables with more than 20% missingness were treated as “unknown”. ALK: alkaline phosphatase; ctDNA TF: circular tumor DNA Tumor Fraction; ECOG PS: Eastern Cooperative Oncology Group performance score; ER: estrogen receptor; IDC: invasive ductal carcinoma; ILC: invasive lobular carcinoma; LLN = lower limit of normal; LDH: NLR: neutrophil-to-lymphocyte ratio; lactate dehydrogenase; PR: progesterone receptor ULN = upper limits of normal.

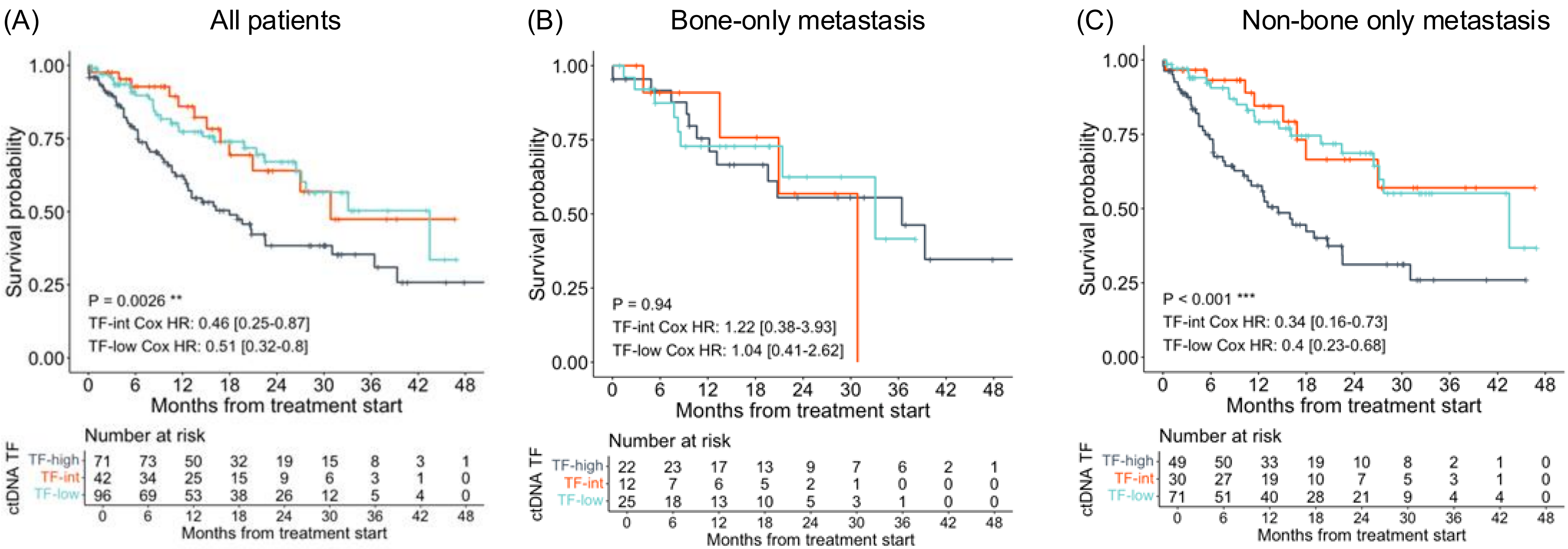

(A)

TF_group	n	events	median	95% CI
TF-high	112	54	17.97	12.62-31.05
TF-int	46	12	30.85	20.93-NR
TF-low	98	28	43.47	27.14-NR

(B)

TF_group	n	events	median	95% CI
TF-high	29	12	36.44	19.58-NR
TF-int	12	4	30.85	20.93-NR
TF-low	26	8	33.08	21.39-NR

(C)

TF_group	n	events	median	95% CI
TF-high	83	42	14.46	10.64-22.54
TF-int	34	8	NR	17.94-NR
TF-low	72	20	43.47	27.14-NR

Supp Figure S3. Real-world overall survival (unadjusted) for metastatic breast cancer patients by ctDNA TF status (1st line of therapy).
(A) All patients; (B) patients with bone-only metastasis; (C) patients with non-bone only metastasis
CI: confidence interval; ctDNA TF: circulating tumor DNA Tumor Fraction; HR: hazard ratio; NR: not reached.

Variable		N	Events	Estimate (95% CI)	p-value
**ctDNA TF**	TF-low	298	99	Reference	
	TF-int	175	81	1.36 (1.00, 1.84)	0.050
	TF-high	304	175	1.85 (1.40, 2.45)	<0.001
**Age**		777		1.02 (1.00, 1.03)	0.032
**ECOG PS**	0	307	117	Reference	
	1	370	172	1.14 (0.89, 1.47)	0.310
	2+	100	66	2.18 (1.53, 3.10)	<0.001
**Race/Ethnicity**	Not Hispanic or Latino, white	421	190	Reference	
	Not Hispanic or Latino, non-white	161	67	1.01 (0.75, 1.35)	0.961
	Hispanic or Latino	37	12	0.78 (0.43, 1.41)	0.404
	Unknown race and/or ethnicity	158	88	1.23 (0.94, 1.62)	0.134
**ER**	Negative	168	90	Reference	
	Positive	609	265	0.48 (0.35, 0.65)	<0.001
**PR**	Negative	357	171	Reference	
	Positive	420	184	1.06 (0.82, 1.38)	0.649
**HER2**	Negative	695	321	Reference	
	Positive	82	34	0.68 (0.47, 0.99)	0.045
**Histology**	IDC	392	176	Reference	
	ILC	67	38	1.40 (0.95, 2.04)	0.086
	Other/Unknown	318	141	0.88 (0.50, 1.53)	0.647
**Line of therapy**	1	256	94	Reference	
	2	165	80	1.60 (1.16, 2.19)	0.004
	3	114	54	1.44 (0.99, 2.08)	0.055
	4+	242	127	1.71 (1.27, 2.30)	<0.001
**Practice type**	Academic/Community	116	46	Reference	
	Community only	661	309	1.07 (0.74, 1.55)	0.721
**Stage at Dx**	Stage I	118	54	Reference	
	Stage II	207	101	0.77 (0.54, 1.09)	0.146
	Stage III	162	72	0.76 (0.53, 1.09)	0.139
	Stage IV	223	97	1.22 (0.65, 2.28)	0.540
	Unknown/not documented	67	31	1.56 (0.79, 3.08)	0.196
**Adjuvant Tx**	No adj tx / unknown	452	197	Reference	
	Adjuvant Tx	325	158	1.49 (1.10, 2.02)	0.010
**Menopausal status**	Premenopausal	36	11	Reference	
	Male/unknown	161	73	1.01 (0.52, 1.96)	0.970
	Postmenopausal	580	271	0.86 (0.43, 1.72)	0.663
**Number of mets**	1	294	94	Reference	
	2+	483	201	1.81 (1.25, 2.63)	0.002
**Metastasis site**	Bone only	155	57	Reference	
	Non-bone only	622	298	0.95 (0.61, 1.48)	0.814
**Albumin**	>=LLN	672	277	Reference	
	<LLN	105	78	2.16 (1.59, 2.93)	<0.001
**ALK**	=<ULN	555	223	Reference	
	>ULN	222	132	1.31 (1.01, 1.69)	0.038
**Serum creatinine**	=<ULN	672	299	Reference	
	>ULN	105	56	1.29 (0.94, 1.76)	0.116
**Hemoglobin**	>=LLN	438	161	Reference	
	<LLN	339	194	1.42 (1.13, 1.78)	0.003
**LDH**	=<ULN	82	40	Reference	
	>ULN	85	38	1.00 (0.62, 1.61)	0.988
	Unknown	610	277	1.05 (0.74, 1.49)	0.781
**NLR**	=<2.5	230	83	Reference	
	>2.5	378	194	1.44 (1.09, 1.89)	0.011
	Unknown	169	78	1.26 (0.88, 1.80)	0.206

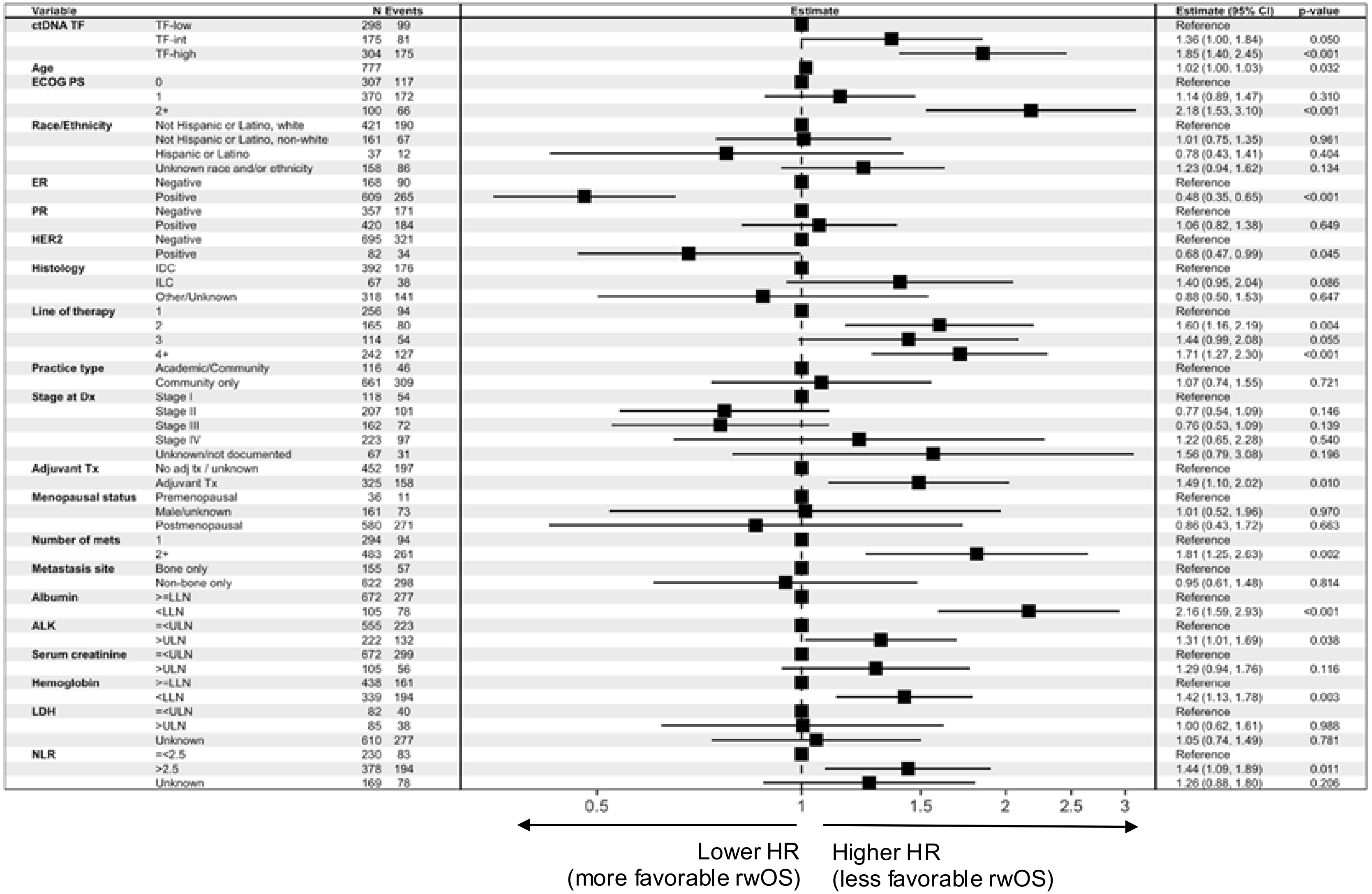

Supp Figure S4. Multivariable model for clinical and laboratory factors associated with real-world overall survival for metastatic breast cancer patients.

ALK: alkaline phosphatase; ctDNA TF: circular tumor DNA Tumor Fraction; ECOG PS: Eastern Cooperative Oncology Group performance score; ER: estrogen receptor; IDC: invasive ductal carcinoma; ILC: invasive lobular carcinoma; LLN = lower limit of normal; LDH: NLR: neutrophil-to-lymphocyte ratio; lactate dehydrogenase; PR: progesterone receptor ULN = upper limits of normal.

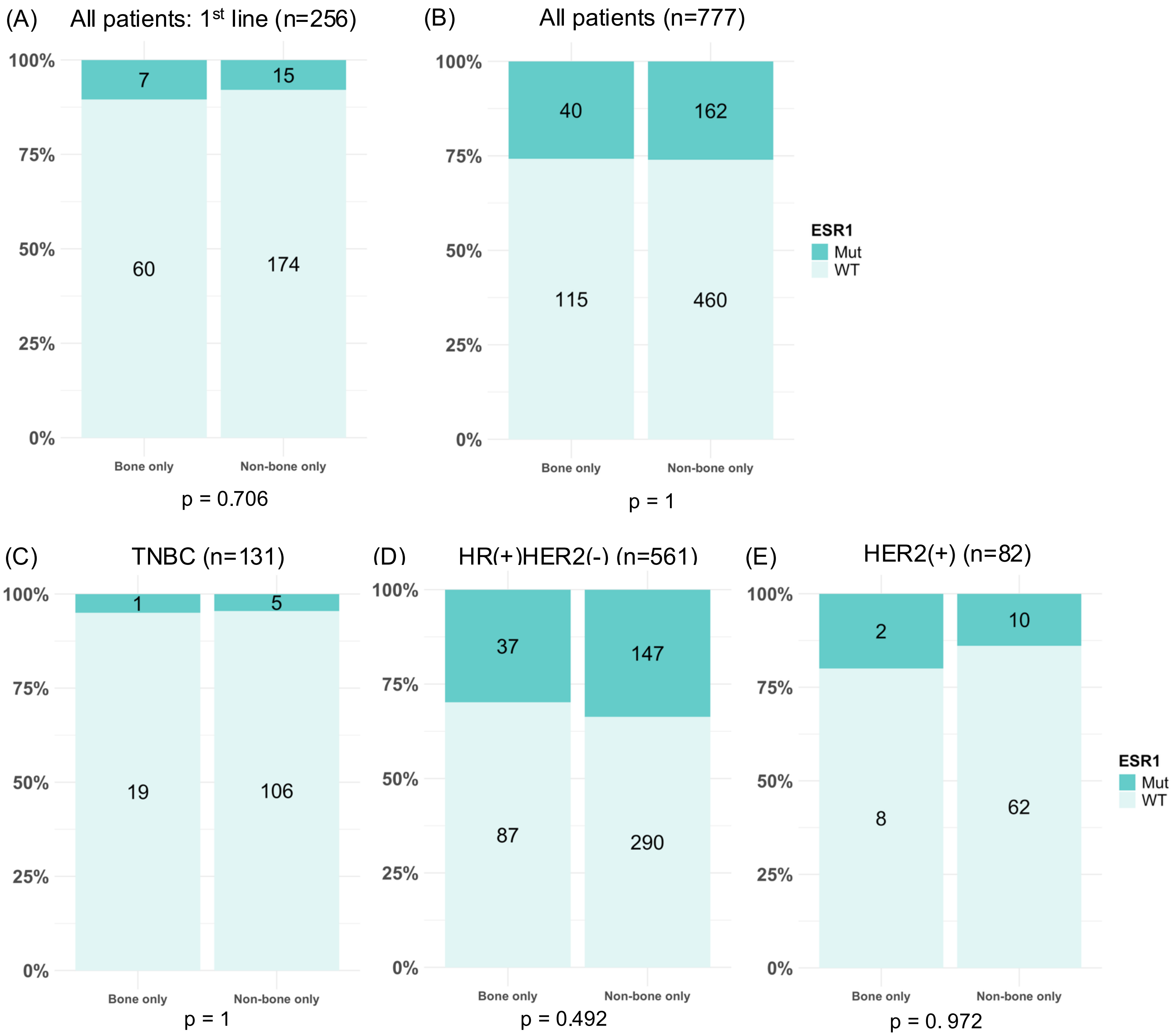

Supp Figure S5. ESR1 mutations distribution in patients with bone-only vs. non-bone only metastatic breast cancer (MBC).
(A) All patients, restricted to samples collected at first line; (B) All patients; (C) Patients with triple-negative breast cancer (TNBC), defined as estrogen and progesterone receptor (hormone receptor/HR) negative and HER2-negative; (D) Patients with HR-positive/HER2-negative MBC; (E) Patients with HER2-positive MBC.

Variable		N	Events	Estimate (95% CI)	p-value
**ctDNA TF**	TF-low	298	99	Reference	
	TF-int	175	81	1.48 (1.08, 2.02)	0.01411
	TF-high	304	175	2.20 (1.65, 2.93)	< 0.001
**Age**		777		1.02 (1.00, 1.03)	0.01460
**ECOG PS**	0	302	115	Reference	
	1	369	172	1.31 (1.03, 1.68)	0.03036
	2+	106	68	3.17 (2.27, 4.41)	< 0.001
**Race/Ethnicity**	Not Hispanic or Latino, white	421	190	Reference	
	Not Hispanic or Latino, non-white	161	67	0.97 (0.73, 1.30)	0.85868
	Hispanic or Latino	37	12	0.67 (0.37, 1.21)	0.18357
	Unknown race and/or ethnicity	158	86	1.33 (1.02, 1.74)	0.03302
**ER**	Negative	168	90	Reference	
	Positive	609	265	0.48 (0.35, 0.65)	< 0.001
**PR**	Negative	357	171	Reference	
	Positive	420	184	0.96 (0.74, 1.24)	0.75509
**HER2**	Negative	695	321	Reference	
	Positive	82	34	0.67 (0.46, 0.97)	0.03335
**Histology**	IDC	392	176	Reference	
	ILC	67	38	1.51 (1.03, 2.21)	0.03363
	Other/Unknown	318	141	0.99 (0.57, 1.72)	0.97169
**Line of therapy**	1	256	94	Reference	
	2	165	80	1.60 (1.17, 2.19)	0.00359
	3	114	54	1.42 (0.99, 2.05)	0.05770
	4+	242	127	1.96 (1.45, 2.65)	< 0.001
**Practice type**	Academic/Community	116	46	Reference	
	Community only	661	309	1.00 (0.72, 1.39)	0.99100
**Stage at Dx**	Stage I	118	54	Reference	
	Stage II	207	101	0.85 (0.60, 1.20)	0.35607
	Stage III	162	72	0.76 (0.53, 1.10)	0.14717
	Stage IV	223	97	1.01 (0.54, 1.89)	0.96701
	Unknown/not documented	67	31	1.24 (0.63, 2.43)	0.52876
**Adjuvant Tx**	No adj tx / unknown	415	180	Reference	
	Adjuvant Tx	362	175	1.22 (0.88, 1.68)	0.23733
**Menopausal status**	Premenopausal	36	11	Reference	
	Male/unknown	161	73	1.11 (0.58, 2.15)	0.74759
	Postmenopausal	580	271	0.86 (0.43, 1.72)	0.67003
**Number of mets**	1	294	94	Reference	
	2+	483	261	2.14 (1.48, 3.09)	< 0.001
**Metastasis site**	Bone only	155	57	Reference	
	Non-bone only	622	298	0.91 (0.59, 1.42)	0.68764
**ESR1**	WT	575	246	Reference	
	Mut	202	109	0.84 (0.65, 1.10)	0.20049

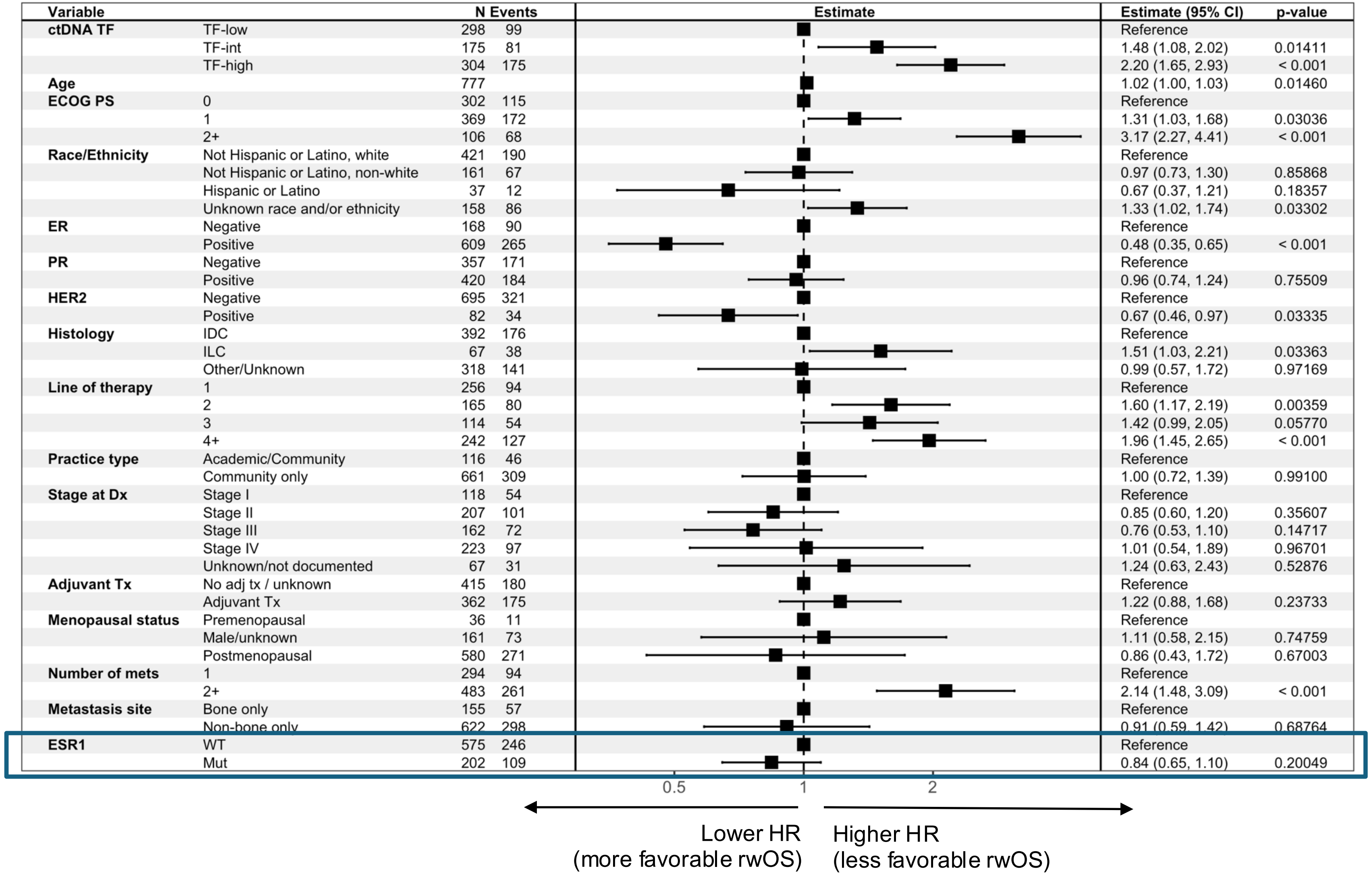

Supp Figure S6. Multivariable model for clinical features including ESR1mut associated with real-world overall survival for metastatic breast cancer patients.
ALK: alkaline phosphatase; ctDNA TF: circular tumor DNA tumor fraction; ECOG PS: Eastern Cooperative Oncology Group performance score; ER: estrogen receptor; IDC: invasive ductal carcinoma; ILC: invasive lobular carcinoma; LLN = lower limit of normal; LDH: NLR: neutrophil-to-lymphocyte ratio; lactate dehydrogenase; PR: progesterone receptor ULN = upper limits of normal.
